# Characterization of IFNγ-producing natural killer cells induced by cytomegalovirus reactivation after haploidentical hematopoietic stem cell transplantation

**DOI:** 10.18632/oncotarget.13916

**Published:** 2016-12-12

**Authors:** Fengyan Jin, Hai Lin, Sujun Gao, Hengxiang Wang, Hongmin Yan, Jinglong Guo, Zheng Hu, Chunhui Jin, Yongqi Wang, Zhidong Wang, Yangzhi Zhao, Yu Liu, Xiaoli Zheng, Yehui Tan, Wei Li, Yun Dai, Yanping Yang

**Affiliations:** ^1^ Department of Hematology, Cancer Center, the First Bethune Hospital of Jilin University, Changchun, Jilin, China; ^2^ Department of Hematology, Air Force General Hospital, the Chinese People's Liberation Army, Beijing, China; ^3^ Institute of Translational Medicine, the First Bethune Hospital of Jilin University, Changchun, Jilin, China

**Keywords:** NK cells, NKG2C, KIR, IFNγ, cytomegalovirus, Pathology Section

## Abstract

During human cytomegalovirus (CMV) infection after umbilical cord blood or HLA-matched hematopoietic stem cell transplantation (HSCT), a population of NKG2C-expressing natural killer (NK) cells expand and persist. The expanded NK cells express high levels of inhibitory killer immunoglobulin-like receptors (KIR) specific for self-HLA and potently produce IFNγ. However, it remains unknown whether similar events would occur after haploidentical HSCT (haplo-HSCT). Here, we demonstrated that IFNγ-producing NK cells were expanded in haplo-HSCT patients with CMV reactivation. We then identified these expanded cells as a subset of CD56^dim^ NK cells that expressed higher levels of both NKG2C and KIR, but lower level of NKG2A. Functionally, the subset of NK cells expressing NKG2C and self-KIR in patients with CMV reactivation accounted for IFNγ production in response to K562 cells. However, these phenomena were not observed in patients without CMV reactivation. We therefore characterized a subset of NK cells with the CD56^dim^, NKG2C^+^, and self-KIR^+^ phenotype that expanded and were responsible for IFNγ production during CMV infection after haplo-HSCT. Together, these findings support a notion that CMV reactivation induces expansion of more mature NK cells with memory-like features, which contributes to long-term control of both CMV infection and leukemia relapse after haplo-HSCT.

## INTRODUCTION

Allogeneic hematopoietic stem cell transplantation (HSCT) is a well-established and life-saving treatment for many patients with cancer, particularly hematological malignancies [1]. While it is ideal to perform HSCT using cells from an human leukocyte antigen (HLA)-matched related donor (MRD) such as HLA-identical sibling, about two-thirds of patients would however have no MRD available for HSC donation [2]. Thus, haploidentical HSCT (haplo-HSCT), with the advantage of more choice in donor selection, offers an alternative approach for treatment of patients with advanced leukemia, particularly those who have neither MRD and HLA-matched unrelated donoror umbilical cord blood available for transplantation [2].

NK cells, as the first population of lymphocytes that reconstitute in recipients after HSCT, play a pivotal role in defense against infections and leukemia relapse, two major challenges for success of haplo-HSCT. The function of NK cells is regulated by diverse surface receptors that transmit either activating or inhibitory signals into NK cells [3–5]. Accordingly, the surface receptors on NK cells include the activating and inhibitory forms of the C-type lectin-like family receptors (NKG2s) and the killer cell immunoglobulin (Ig)-like receptors (KIRs). In normal individuals, both activating and inhibitory receptors on NK cells are involved in killing of virus-infected cells or tumor cells *via* the corresponding signals triggered by binding of the specific their cognate ligands, class I HLA molecules [6]. After binding to the specific ligands on target cells, while the inhibitory receptors prevent the cytotoxic action of NK cells, stimulation of the activating receptors mediates killing of target cells by NK cells [7]. Of note, whereas primary human CMV infection usually occurs asymptomatically, virus infection could be a potentially life-threatening complication in patients with immunodeficiency, e.g., transplantation recipients [8]. Responses to cytomegalovirus (CMV) infection lead to stable imprints in the KIR repertoire of human NK cells [9]. Moreover, latent CMV infection induces a permanent up-regulation of the activating receptor NKG2C [10, 11], in association with modulation of the NK cell KIR repertoire [12, 13]. Recently, increasing evidence indicates that NK cells also exhibit memory-like properties comparable to B and T lymphocytes [14, 15].

Following CMV reactivation in patients who have received CMV-naive umbilical cord blood or MUD HSCT, a subset of reconstituting NK cells expand and display an increased density of surface NKG2C [10, 11]. Interestingly, these NK cells often persist for long term (e.g., a year after transplantation) even after viral clearance, indicating their memory-like features. Moreover, they have been characterized by predominant expression of NKG2C and the inhibitory KIR specific for self-HLA, but lack of NKG2A, a phenotype required for robust IFNγ production [10]. However, it remains unknown whether similar CMV-induced events also occur in patients after haplo-HSCT that often causes delayed immune reconstitution due to more severe immunosuppression than HLA-matched HSCT. The aim of our study was to determine and characterize NK cells that expand and function to produce IFNγ during CMV reactivation in patients with hematologic malignancies who have received the treatment of haplo-HSCT.

## RESULTS

### IFNγ-producing NK cells expand in response to CMV reactivation in haplo-HSCT patients

**Figure 1:**
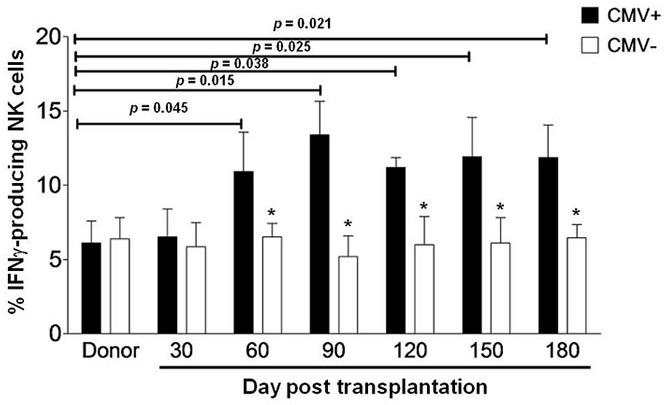
IFNγ-producing NK cells expand in haplo-HSCT patients with CMV reactivation PBMCs from the donors before haplo-HSCT (*n* = 10) and the haplo-HSCT patients who had acute GVHD 0-1 after haplo-HSCT (*n* = 29) were collected and analyzed by flow cytometry at days 30, 60, 90, 120, 150 and 180 after transplantation. Cells were then incubated with K562 cells for 5 hrs, after which the percentage of NK cells producing IFNγ was measured in the CD56^+^/CD3^−^ subset. IFNγ production was measured in 19 patients with CMV reactivation (CMV^+^) and 10 patients without CMV reactivation (CMV^−^). Values represent mean ± SEM. *P < 0 .05 for comparisons between the CMV^+^ and CMV^−^ samples.

Recent studies have demonstrated that the expansion of IFNγ-producing NK cells is specifically associated with CMV infection in patients after umbilical cord blood and HLA-matched allogeneic HSCT [10, 11]. We thus first examined whether similar event also occurs in patients after haplo-HSCT. As NK cell counts are significantly different between patients who had grades 2-4 acute graft-*versus*-host disease (GVHD) *versus* grades 0-1 within six months after transplantation, we examined the percentage of IFNγ-producing NK cells only in patients who had grades 0-1 GVHD after haplo-HSCT, in order to avoid such effects of GVHD. The clinical characteristics for these patients with hematologic malignancies were summarized in Table 1. CMV reactivation was monitored by qPCR twice a week in all patients. The antiviral therapy was given when CMV was detected, and CMV became undetectable in the blood after 2 to 4 week treatment. In a total of 29 patients, 19 had CMV reactivation, while 10 were CMV-seronegative. Because target cell-induced IFNγ production of NK cells in recipients of unmanipulated or CD34^+^ selected grafts usually drops to the basal levels in normal donors after 6 months post HSCT [16], we therefore decided to carry out all of the analyses within the first 180 days after haplo-HSCT. To this end, peripheral blood mononuclear cells (PBMCs) were collected from each patient at day 30, 60, 90, 120, 150, and 180 after haplo-HSCT. Notably, all 19 patients with CMV reactivation displayed expansion of IFNγ-producing NK cells, in whom the percentages of IFNγ-producing NK cells were significantly increased from day 60 to 180 after transplantation, compared to those for their donors (day 60, 10.93 ± 2.64 *versus* 6.13 ± 1.46, *P* = 0.045; day 90, 13.42 ± 2.26 *versus* 6.13 ± 1.46, *P* = 0.015; day 120, 11.23 ± 0.61 *versus* 6.13 ± 1.46, *P* = 0.038; day 150, 11.98 ± 2.46 *versus* 6.13 ± 1.46, *P* = 0.025; day 180, 11.76 ± 2.19 *versus* 6.13 ± 1.46, *P* = 0.021; Figure 1). This NK cell response started at day 60, with a maximum at day 90, after transplantation. In sharp contrast, there was no increase in the percentage of IFNγ-producing NK cells in patients without CMV reactivation (*P* > 0.05 for all time points, compared to those for their donors, while *P* < 0.05 for comparisons between patients with or without CMV reactivation after 60 days post transplantation). These findings indicate that as previously observed in the case of umbilical cord blood or HLA-matched allogeneic HSCT, the expansion of IFNγ-producing NK cells also occurs in CMV-seropositive, but not -seronegative, patients with after haplo-HSCT.

**Table 1:** Clinical characteristics of haplo-HSCT patients (n = 29)

Characteristics	Number of patients (n)
Age, median (range)	24 yrs (5 - 45)
Gender	
Male	17
Female	12
Disease type	
Acute myeloid leukemia	2
Acute lymphoblastic leukemia	14
Other hematologic malignancy	3
Risk stratification	
Intermediate	14
high	15
Time of transplantation	
CR1	12
CR2	17
Time after HSCT, median (range)	36 days (19 - 69)
CMV reactivation	
Yes	19
No	10

CR, complete remission

### CMV reactivation induces a more mature phenotype of NK cells after haplo-HSCT

**Figure 2:**
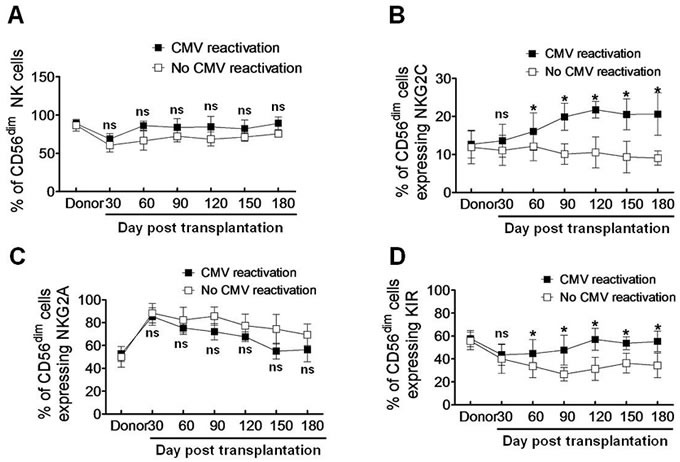
CMV reactivation induces a mature phenotype of NK cells (CD56^dim^) expressing NKG2C and KIR after haplo-HSCT The percentages of CD56^dim^ NK cells **A.**, as well as NK cells expressing NKG2C **B.**, NKG2A **C.** and KIR **D.** in the CD56^dim^ subset were determined by flow cytometry in patients with (■, *n* = 19) without CMV reactivation (□, *n* = 10) at days 30, 60, 90, 120, 150, and 180 after haplo-HSCT. Values represent mean ± SEM. *P < 0 .05 and ns (not significant) *P* > 0.05 for comparisons between the CMV reactivation and no reactivation groups.

We next phenotyped IFNγ-producing NK cells in haplo-HSCT patients with CMV reactivation. NK cell differentiation is featured by CD56 expression on cell surface. In peripheral blood of normal individuals, only approximately 2-10% NK cells display a high density of surface CD56 (designated CD56^bright^), while a majority (~ 90%) of NK cells have a low density of surface CD56 (designated CD56^dim^). While the CD56^dim^ subset of NK cells exhibit limited potential of proliferation, they become functionally active, reflected by KIR expression and an abundance of intracellular cytotoxic granules, in response to *ex vivo* stimulation with interleukin-2 or interleukin-15 [15, 17–19]. We thus analyzed the CD56^dim^ population of NK cells in haplo-HSCT patients with CMV infection. Although a modest increase of the CD56^dim^ NK cell population was observed after day 60 post transplantation, there was no statistically significant difference in the percentages of CD56^dim^ NK cells between patients with and without CMV reactivation at all time points (*P* > 0.05, Figure 2A). As expression of the C-type lectin-like receptor NKG2C that recognizes HLA-E represents a major activating signal in NK cells [20], we further examined expression of NKG2C on CD56^dim^ NK cells. Notably, the percentage of NKG2C-expressing NK cells in the CD56^dim^ subset was significantly higher in haplo-HSCT patients with CMV reactivation, compared to that in patients without CMV reactivation (day 60, 16.03 ± 2.85 *vs* 12.16 ± 2.21, *P* = 0.042; day 90, 19.92 ± 2.04 *vs* 10.13 ± 1.57, *P* = 0.0068; day 120, 21.77 ± 1.27 *vs* 10.53 ± 2.37, *P* = 0.0045; day 150, 20.53 ± 2.35 *vs* 9.33 ± 2.46, *P* = 0.0052; day 180, 20.65 ± 3.22 *vs* 9.06 ± 1.07, *P* = 0.0040; Figure 2B). To exclude a possibility that HLA-E alterations might alter NKG2C expression, another C-type lectin-like receptor NKG2A, which also recognizes HLA-E but acts as an inhibitory signal in NK cells, was monitored in parallel [5]. In contrast to increased NKG2C expression, there was no significant difference in the percentage of NKG2A-expressing CD56^dim^ NK cells between patients with or without CMV reactivation, while instead, a moderate decrease was noted in patients with CMV reactivation although not statistically significant (*P* > 0.05 at each time point; Figure 2C).

Similar to expression of the activating receptor Ly49H in mice after CMV infection [21], KIRs are expressed on NK cells during human CMV reactivation [10, 11], an event required for educating NK cells to produce IFNγ after allogeneic HSCT [22]. In this context, we then examined expression of KIRs on CD56^dim^ NK cells in response to CMV infection after haplo-HSCT. Again, the percentage of KIR-expressing NK cells was significantly higher in haplo-HSCT patients with CMV reactivation than those without CMV reactivation (day 60, 44.64 ± 7.03 *vs* 33.53 ± 5.78, *P* = 0.045; day 90, 47.67 ± 7.51 *vs* 26.67 ± 3.38, *P* = 0.028; day 120, 57.25 ± 5.51 *vs* 31.35 ± 5.76, *P* = 0.015; day 150, 53.62 ± 3.18 *vs* 36.42 ± 4.91, *P* = 0.036; day 180, 55.26 ± 5.17 *vs* 34.32 ± 6.17, *P* = 0.030; Figure 2D). Together, these findings suggest that CMV reactivation induces a more mature phenotype of NK cells featured by CD56^dim^ and expression of NKG2C and KIR after haplo-HSCT.

### Self-KIR is preferentially expressed on the expanded NKG2C^+^ NK cells in response to CMV reactivation after haplo-HSCT

**Figure 3:**
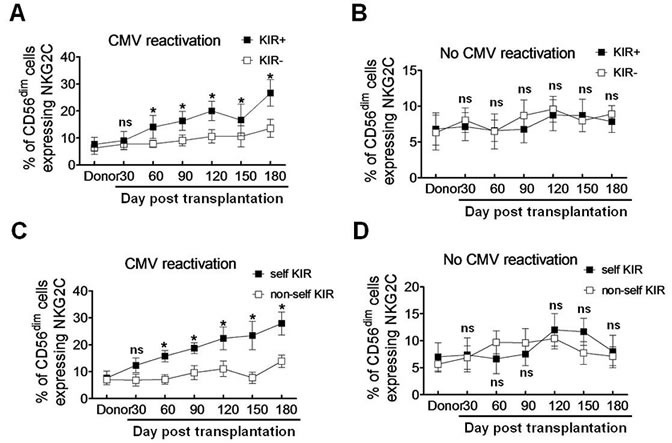
Self-KIR is expressed on expanding NKG2C^+^ NK cells during CMV reactivation post haplo-HSCT **A.**-**B.** KIR^+^ and KIR^−^ NK cells were distinguished by flow cytometry using antibodies specifically against KIR2DL1, KIR3DL1 and KIR2DL3. The percentage of the CD56^dim^ NKG2C^+^ NK cells were determined in the KIR^+^ (■) and KIR^−^(□) populations respectively in patients with (**A.**, *n* = 19) or without CMV reactivation (**B.**, *n* = 10). Values represent mean ± SEM. *P < 0 .05 and ns (not significant) *P* > 0.05 for comparisons between the KIR^+^ and KIR^−^ groups. **C.**-**D.** Self-KIR and non-self-KIR were determined based on the patient KIR ligand status. The percentage of the CD56^dim^ NKG2C^+^ NK cells were assessed by flow cytometry in the self-KIR (■) and non-self-KIR(□) patients with (**C.**, *n* = 10 for self-KIR and *n* = 9 for non-self-KIR) or without CMV reactivation (**D.**, *n* = 5 for self-KIR and non-self-KIR each), respectively. Values represent mean ± SEM. *P < 0 .05 and ns (not significant) *P* > 0.05 for comparisons between the self-KIR and non-self-KIR groups.

As KIR expression is required for educating NKG2C^+^ NK cells to produce IFNγ [10, 11], we then examined whether KIRs are preferentially expressed on NKG2C^+^ NK cells in patients with CMV reactivation after haplo-HSCT. Since CD56^dim^ NKG2C^+^ NK cells were expanded in response to CMV reactivation (Figure 2B), we selected this subset of NK cells for further analyses. There was a significant increase in the percentage of CD56^dim^ NKG2C^+^ NK cells expressing KIR (KIR^+^), compared to that for KIR^−^ cells, in patients with CMV reactivation at each time point after 60 days post transplantation (day 60, 14.06 ± 2.47 *vs* 7.81 ± 0.76, *P* = 0.046; day 90, 16.34 ± 2.02 *vs* 9.03 ± 1.15, *P* = 0.037; day 120, 20.25 ± 2.08 *vs* 10.56 ± 1.43, *P* = 0.032; day 150, 16.67 ± 3.38 *vs* 10.63 ± 2.25, *P* = 0.043; day 180, 26.67 ± 2.84 *vs* 13.56 ± 1.93, *P* = 0.022; Figure 3A). However, such a phenomenon was not observed in haplo-HSCT patients without CMV reactivation (*P* > 0.05 for each time point; Figure 3B).

KIRs specifically recognize the HLA class I ligands that contain four polymorphic epitopes for HLA-A, -B, and -C each. C1 and C2 epitopes of HLA are distinguished from each other by different amino acids at position 80 [23]. C1 epitope recognizes the inhibitory receptors KIR2DL2/3, while C2 recognizes both the inhibitory receptor KIR2DL1 and the activating receptor KIR2DS1. Bw4 and A3/11 epitope of HLA also recognize KIR3DL1 and KIR3DL2, respectively [24–26]. The inhibitory KIR specific for self-MHC I is known as self-KIR, while that not specific for self-MHC I as non-self KIR. The characteristics of KIR and HLA ligands in donors and haplo-HSCT patients were shown in Table 2. Because expanded NKG2C^+^ NK cells might be educated through self-HLA class-I, we next examined whether individual KIR that recognizes self-HLA would be preferentially expressed. In 19 patients with CMV reactivation, 10 patients had self-KIR^+^ NK cells, while 9 patients had non-self KIR^+^ NK cells. The percentage of self-KIR^+^ NK cells in the CD56^dim^ NKG2C^+^ subset was significantly increased, compared to non-self KIR^+^ NK cells, in patients with CMV reactivation after haplo-HSCT (day 60, 15.76 ± 1.22 *vs* 7.13 ± 1.07, *P* = 0.037; day 90, 18.76 ± 1.15 *vs* 9.64 ± 1.51, *P* = 0.041; day 120, 22.37 ± 2.42 *vs* 11.06 ± 1.68, *P* = 0.032; day 150, 23.41 ± 3.03 *vs* 7.74 ± 1.23, *P* = 0.015; day 180, 27.82 ± 2.46 *vs* 13.87 ± 1.34, *P* = 0.025; Figure 3C). Again, there was no significant difference between self-KIR^+^ and non-self KIR^+^ NK cells in patients without CMV reactivation (*P* > 0.05 for each time point; Figure 3D). These findings indicate that the expanded CD56^dim^ NKG2C^+^ NK cells preferentially express KIR, especially self-KIR, in response to CMV reactivation in patients after haplo-HSCT. It is noteworthy that expression of either NKG2C (Figure 2B) or KIR (Figure 2D), as well as both NKG2C and KIR (Figure 3A) or self-KIR (Figure 3C), in the CD56^dim^ subset of NK cells persisted for at least 6 months, suggesting their memory-like features as observed after umbilical cord blood HSCT [10].

**Table 2:** Characteristics of KIR and HLA ligands in haplo-HSCT patients (n = 29)

Patients	Donor KIR	KIR-ligand genotype of patients				Self-KIR	CMV
		HLA-C1	HLA-C2	HLA-Bw4	HLA-A03/11		
1	2DL2	+	−	+	−	Y	+
2	2DL1	+	−	−	+	N	+
3	2DL3	+	−	−	−	Y	+
4	3DL1	−	+	−	−	N	+
5	2DL1	−	+	+	−	Y	+
6	2DL3	−	+	−	−	N	+
7	3DL1	−	+	+	−	Y	+
8	2DL2	−	+	−	−	N	+
9	2DL2	+	−	+	−	Y	+
10	2DL3	+	−	−	−	Y	+
11	3DL1	+	+	−	−	N	+
12	2DL1	+	−	+	−	N	+
13	2DL2	+	+	+	−	Y	+
14	3DL1	+	+	+	−	Y	+
15	2DL1	+	−	−	+	N	+
16	2DL3	+	+	+	−	Y	+
17	2DL2	−	+	+	−	N	+
18	2DL1	+	−	−	−	N	+
19	3DL1	+	−	+	−	Y	+
20	2DL1	−	+	−	+	Y	−
21	3DL1	+	−	+	−	Y	−
22	3DL1	−	+	−	−	N	−
23	2DL3	+	+	−	−	Y	−
24	2DL2	−	+	−	+	N	−
25	2DL1	+	−	+	−	N	−
26	3DL1	−	+	+	−	Y	−
27	2DL3	+	−	−	−	Y	−
28	2DL2	−	+	+	−	N	−
29	2DL1	+	−	−	−	N	−

### NKG2C^+^ NK cells are functional in post haplo-HSCT patients with CMV reactivation

**Figure 4:**
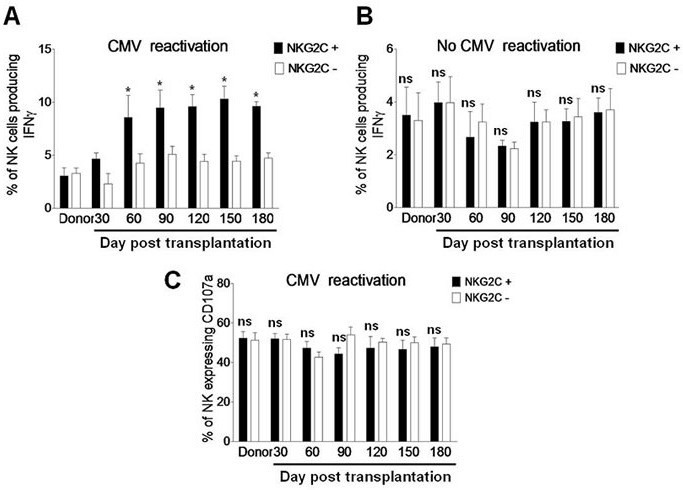
NKG2C is expressed in IFNγ-producing NK cells during CMV reactivation post haplo-HSCT **A.** PBMCs from the donors (*n* = 8) and haplo-HSCT patients with CMV reactivation (*n* = 19) and **B.** from the donors (*n* = 6) and haplo-HSCT patients without CMV reactivation (*n* = 10) were collected at days 30, 60, 90, 120, 150, and 180 after transplantation. Cells were then incubated with K562 cells for 5 hrs, after which the percentages of IFNγ-producing NK cells were determined by flow cytometry in the NKG2C^+^ (■) or NKG2C^−^ (□) subset, respectively. **C.** In parallel, CD107a expression was measured by flow cytometry as positive control. Values represent mean ± SEM. *P < 0 .05 and ns (not significant) *P* > 0.05 for comparisons between the NKG2C^+^ and NKG2C^−^ groups.

In a well-characterized murine model of CMV infection, memory-like NK cells expressing the activating receptor Ly49H recognize the viral protein m157 and expand after the initial viral infection [5]. Of note, similar memory NK-cell response has been demonstrated in human CMV infection [11]. As NKG2C^+^ NK cells expanded and preferentially expressed KIR during CMV reactivation (Figure 3A), we then examined whether the expanded NKG2C^+^ NK cells were functional by measuring IFNγ production and CD107a expression as negative control in response to the HLA class I-negative K562 cells [10]. After incubation with K562 cells for 5 hrs, the percentage of IFNγ-producing NKG2C^+^ NK cells was significantly greater than that for NKG2C^−^ NK cells in patients with CMV reactivation after haplo-HSCT (day 30, 4.63 ± 0.57 *vs* 2.32 ± 0.56, *P* = 0.072; day 60, 8.56 ± 2.06 *vs* 4.23 ± 0.87, *P* = 0.036; day 90, 9.46 ± 1.67 *vs* 5.15 ± 0.72, *P* = 0.040; day 120, 9.57 ± 1.14 *vs* 4.42 ± 0.73, *P* = 0.028; day 150, 10.31 ± 1.19 *vs* 4.43 ± 0.49, *P* = 0.023; day 180, 9.61 ± 0.45 *vs* 4.72 ± 0.52, *P* = 0.035; Figure 4A). However, there was no significant difference in the percentage of IFNγ-producing NK cells between the NKG2C^+^ and NKG2C^−^ subsets in patients without CMV reactivation (*P* > 0.05 for each time point; Figure 4B). Moreover, there was also no significant difference in the percentage of NK cells expressing CD107a between the NKG2C^+^ and NKG2C^−^ subsets in patients with CMV reactivation (*P* > 0.05 for each time point; Figure 4C). These findings suggest that NKG2C^+^ NK cells in haplo-HSCT patients with CMV reactivation are functional for long-term to produce IFNγ.

### Self-KIR expression on NK cells of post haplo-HSCT patients with CMV reactivation is required for IFNγ production

**Figure 5:**
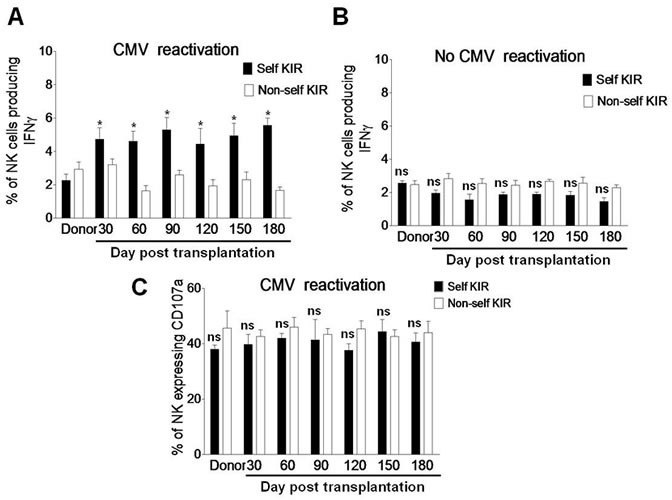
Self-KIR is expressed in IFNγ-producing NK cells during CMV reactivation post haplo-HSCT **A.** PBMCs from the donors (*n* = 8) and haplo-HSCT patients with CMV reactivation (*n* = 19) and **B.** from the donors (*n*= 6) and haplo-HSCT patients without CMV reactivation (*n* = 10) were collected at days 30, 60, 90, 120, 150, and 180 after transplantation. Cells were then incubated with K562 cells for 5 hrs, after which the percentages of IFNγ-producing NK cells were determined by flow cytometry in the self-KIR (■) or non-self KIR (□) subset. **C.** In parallel, CD107a expression was measured by flow cytometry as positive control. Values represent mean ± SEM. *P < 0 .05 and ns (not significant) *P* > 0.05 for comparisons between the self-KIR and non-self-KIR groups.

The functions of NK cells reconstituted after transplantation include cytokine production and degranulation. Whereas degranulation is similar between the KIR^+^ and KIR^−^ populations, target cell-induced IFNγ production is restricted only to the subset of KIR^+^ NK cells [22]. In this context, we last examined whether the expanded self-KIR^+^ NK cells are functional by measuring IFNγ production and CD107a expression in response to K562 cells as described above. After exposure to K562 cells for 5 hrs, the percentage of IFNγ-producing NK cells was significantly increased in the subset of self-KIR^+^ NK cells, but not non-self KIR^+^ NK cells, in patients with CMV reactivation after haplo-HSCT (day 30, 4.73 ± 0.68 *vs* 3.22 ± 0.36, *P* = 0.046; day 60, 4.63 ± 0.61 *vs* 1.63 ± 0.318, *P* = 0.022; day 90, 5.32 ± 0.73 *vs* 2.62 ± 0.26, *P* = 0.028; day 120, 4.43 ± 0.93 *vs* 1.93 ± 0.37, *P* = 0.039; day 150, 4.93 ± 0.76 *vs* 2.32 ± 0.46, *P* = 0.032; day 180, 5.56 ± 0.43 *vs* 1.66 ± 0.23, *P* = 0.021; Figure 5A). However, between the self-KIR^+^ and non-self KIR^+^ subsets, there was no significant difference in the percentage of IFNγ-producing NK cells in patients without CMV reactivation (Figure 5B, *P* > 0.05 for each time point) or CD107a-expressing NK cells as negative control in patients with CMV reactivation (Figure 5C, *P* > 0.05 for each time point). These findings suggest that self-KIR^+^ NK cells in patients with CMV infection after haplo-HSCT are functional for long term to produce IFNγ.

## DISCUSSION

In the present study, we demonstrated that IFNγ-producing NK cells expanded in response to CMV infection in patients and persisted for at least 6 months after haplo-HSCT, suggesting their memory-like feature. Further, CMV reactivation induced a more mature phenotype of NK cells characterized by CD56^dim^ and preferential expression of NKG2C and self-KIR after transplantation, which are responsible for IFNγ production. Last, NK cells expressing NKG2C and self-KIR in haplo-HSCT patients with CMV reactivation are functional for long term to produce IFNγ in response to K562 cells. Allogeneic HSCT, which subsequently induces a potent graft *versus* leukemia effect, is a potentially curative therapy for hematologic malignancies. While MRD or MUD could not be identified as an ideal donor for a majority of patients, haplo-HSCT with high donor availability appears to offer an immediate treatment option. This approach is also less time-consuming than conventional HSCT that requires a stringent matching process. However, although haplo-HSCT could reduce leukemia relapse, it is also often associated with serious immunosuppression due to treatment of severe GVHD caused by HLA-mismatch and thus increases risk of infection, particularly with CMV. High morbidity and mortality of CMV infection as well as delayed immune reconstitution post transplantation remain to be the critical obstacles for success of haplo-HSCT. However, our study demonstrated that CMV reactivation induces the expansion of cytotoxic NK cells with memory-like features, which might in turn contribute to control of viral infection, as well as probably prevention of leukemia relapse as well, in patients after haplo-HSCT.

It has been reported that there is no statistically difference in counts of total and CD56^dim^ NK cells between patients with malignant or non-malignant diseases within six months after transplanted with either bone marrow or peripheral blood stem cells, and between patients conditioned with either myeloablative and reduced intensity conditioning [27]. However, total and CD56^dim^ NK cells are significantly lower in patients who had grades 2-4 acute GVHD than those with grades 0-1 acute GVHD, indicating acute GVHD as an important determinant that influences counts of total as well as CD56^dim^ NK cells. Therefore, we selected a cohort of patients with 0-1 acute GVHD after haplo-HSCT in this study, in order to avoid the potential effects of acute GVHD on the readouts of our analyses on total and particularly CD56^dim^ NK cells.

In concordance to the previous observation after umbilical cord blood (UCB) and 1 HLA-matched sibling transplantation [10], we observed that CMV reactivation induced expansion of the NKG2C^+^ memory-like NK cells in patients after haplo-HSCT, which in turn produced IFNγ to control of CMV infection. NK cells are the first cell population to reconstitute following HSCT. It has been found that similar to Ly49H^+^ NK cells in mice, NK cells expressing the activating C-type lectin-like receptor NKG2C might represent memory-like NK cells in humans [11, 28]. NKG2C recognizes the non-classical class I allele HLA-E, while its inhibitory counterpart NKG2A also recognizes HLA-E [29]. Although CMV infection results in a down-regulation of the class I HLA, HLA-E often remains intact on the cell surface [30, 31]. Whereas NKG2C^+^ NK cells expand in co-culture with cell lines transfected with HLA-E and IL-15 [32], CMV-infected cells might express HLA-E loaded with a viral peptide that drives NKG2C expansion, or alternatively, encode a viral protein that binds to NKG2C [11]. Moreover, it has been demonstrated that the CD94/NKG2C/HLA-E axis represents an important mechanism for the expansion of NKG2C^+^ NK cells in response to human CMV infection [8]. It has been reported that several toll-like receptors (e.g., TLR2, 3, 9, and recently TLR 5) are involved in NK cell-mediated control of CMV in mice [33, 34]. However, the intracellular signals and molecular mechanism responsible for CMV-induced expansion of NKG2C^+^ NK cells remains virtually unknown in human, especially after HSCT.

The bulk of evidence have demonstrated the relationship between KIR and CMV reactivation. For example, the activating receptor Ly49H in mice, analogous to KIRs in humans, are mainly engaged in control of murine CMV infection [21]. Whereas KIRs are expressed on NK cells during CMV reactivation [10, 11], KIR expression is required for educating NK cells to produce IFNγ after allogeneic HSCT [22]. In the present study, we found that NK cells expressing KIR that recognizes self-HLA, expanded and produced IFNγ in haplo-HSCT patients with CMV reactivation. This result is similar to the previous observations that self-KIR, especially CD158b (e.g., KIR2DL2, KIR2DL3, and KIR2DS2), is predominately expressed on IFNγ-producing NK cells in patients after umbilical cord blood, MRD, or MUD HSCT [10, 11, 22]. We further found that self-KIR was preferentially expressed on expanded NKG2C^+^ NK cells in the present setting, consistent with the earlier observation that CMV drives expansion of NKG2C^+^ cells expressing self-specific KIRs in chronic hepatitis patients [35]. In turn, KIR expression on NK cells is critical for robust IFNγ production, a potential mechanism underlying both control of viral infection and suppression of tumor growth after HSCT [10, 11, 36–38]. Regarding the ligands of KIR, it has been reported that a hierarchical effect on NK cell function primarily depends on different HLA-C1 alleles, but there is no difference between HLA-C2 alleles [39]. Interestingly, donors who are homozygous for HLA-C*07 (HLA-C1) are potent IFNγ producers, while those who are homozygous for HLA-C*1402 are, however, very poor IFNγ producers. On the other hand, expression of the inhibitory KIR specific for self-HLA class I ligand is also essential for alloreactivity of NK cells between donor and recipient [40]. The inhibitory KIR in recipients could sense the missing expression of donor KIR ligands and thereby trigger alloreactions. In this context, KIR2DL1, KIR2DL2/KIR2DL3, and KIR3DL1 recognize the HLA-C2, HLA-C1, and HLA-Bw4 alleles, respectively. Mismatch of these three KIR ligands (in the GvH direction) can lead to donor *versus* recipient NK cell alloreactivity. However, it remains to be defined whether CMV-induced KIR expression might also contribute to alloreactivity of NK cells after HSCT.

In summary, our study revealed expansion of IFNγ-producing NK cells in patients with CMV infection after haplo-HSCT and characterized these expanded cells as a CD56^dim^ subset of NK cells with a more mature phenotype preferentially expressing NKG2C and self-KIR. These results suggest that CMV reactivation might shape the NK cell receptor repertoires. Moreover, NK cells expressing NKG2C and self-KIR during CMV reactivation are functionally responsible for robust IFNγ production as well as display memory-like features. Thus, this study provide evidence supporting a notion that NK cells characterized by CD56^dim^, NKG2C^+^ and self-KIR^+^ expand and persist in response to CMV reactivation in patients after haplo-HSCT, which in turn produce IFNγ to control CMV infection as well as prevent leukemia relapse. Of note, the similarity in the observations between haplo-HSCT in the present study and UCB or MRD HSCT [10] argues strongly that such a NK cell response might solely stem from CMV infection, while independent of HSCT donor types. Therefore, the molecular mechanism(s) by which CMV induces the selective expansion of functional and memory-like NK cells warrants further investigation in patients after HSCT.

## MATERIALS AND METHODS

### Patients

Patient samples were obtained with the approval of Institutional Review Boards and the informed consent from each patient. A total of 29 haplo-HSCT patients were enrolled into this study in the General Hospital of the Air Force of the Chinese People's Liberation Army and the First Bethune Hospital of Jilin University between August 2011 and November 2014. Among them, there were 19 patients with CMV infection and 10 patients without CMV infection. All patients had received myeloablative conditioning with cytarabine (4 g/m ^2^ per day, days −10 to −9), busulfan (3.2 mg/kg per day, intravenously days −8 to −6), cyclophosphamide (1.8 g/m ^2^ per day, days −5 to −4), semustine (250 mg/m ^2^, day −3), and rabbit ATG (thymoglobulin, 2.5 mg/kg per day, days −5 to −2) [41]. All patients were infused with granulocyte colony stimulating factor (G-CSF)-mobilized peripheral blood stem cells (PBSCs) and bone marrow grafts from haploidentical donors (days +1 to +2). GVHD prophylaxis included cyclosporine A, methotrexate, and mycophenolate mofetil. There was no difference in GVHD prophylaxis between patients with or without CMV reactivation. Patients were monitored weekly for CMV reactivation by quantitative PCR (qPCR) performed in the Clinical Virology Laboratory. CMV viremia (> 100 copies) was treated with ganciclovir until CMV viremia became negative (< 100 copies) [11]. Peripheral blood mononuclear cells (PBMCs) from pre-HSCT samples of all donors and post-HSCT samples of recipients at 3 and 6 month after transplantation were collected. After haplo-HSCT, 19 patients developed detectable HCMV reactivation in the blood during 19 - 69 days after transplantation. High resolution HLA typing was performed, by which NK ligand status was assigned on the basis of Bw4, HLA-C1, HLA-C2, and HLA-A03/11 group ligands.

### Antibodies and flow cytometry

The NK cell surface phenotype was determined by flow cytometry (FACSCalibur, BD Biosciences) using the monoclonal antibodies (mAbs) as follows: CD3-PerCP (SK7; BD Biosciences), CD56-allophycocyanin (B159; BD Biosciences), NKG2C-PE (134591; R&D Systems), NKG2A (Z199; Beckman Coulter), NKG2D (ID11; BD Biosciences), KIR2DL1-FITC (143211; R&D Systems), KIR3DL1 (DX9, Miltenyi), KIR3DL2-biotin (streptavidin-Qdot.605), KIR2DL2/2DL3/2DS2-PE (GL183; Beckman Coulter), KIR2DL2/S2/L3 (DX27, Miltenyi), and isotypematched controls (IgG1 from BD Pharmingen, IgG2a from R&D Systems). Dead cells were excluded by propidium iodine (PI) staining, while PI-negative live cells were gated in for analysis. Non-reactive allophycocyanin-labeled rat IgG2a mAbs were used as negative controls.

Intracellular production of IFNγ and expression of CD107a was measured using Pacific blue-conjugated anti-IFNγ (clone 4S.B3; BioLegend) and anti-CD107a (H4A3; BD Biosciences) as reported previously [10]. Briefly, PBMCs were isolated from each sample by density-gradient centrifugation and cryopreserved. Before analysis, cells were thawed and assessed after incubation with K562 cells for 5 h at 37°C in complete DMEM (without exogenous cytokines) supplemented with 20% human AB serum (Valley Biomedical), 30% Ham F-12 medium (Cellgro), 100 U/mL of penicillin (Invitrogen), 100 U/mL of streptomycin (Invitrogen), 24 μM 2-β-mercaptoethanol, 50 μM ethanolamine, 20 mg/L of ascorbic acid, and 50 μg/L of sodium selenate. The human erythroleukemia cell line K562 was maintained in IMDM supplemented with 10% FBS, 100 U/mL of streptomycin, and 100 U/mL of penicillin. Data were analyzed using FlowJo 7.6.1 software (Tree Star) [12].

### HLA and KIR typing

Complete high-resolution, allele-level typing data for HLA-A, HLA-B, HLA-C, HLA-DRB1 and HLA-DQB1 were obtained from the High-resolution HLA Typing Laboratory in the China Marrow Donor Program. KIR genes were typed using the PCR-SSP methods as per the manufacturers' instruction, as described previously [36, 42].

### Statistical analysis

Data are presented as mean and SE (mean ± SEM). Differences between groups were analyzed using the Student's t test. Paired t test was used for comparisons of matched samples. All statistical analyses were performed using SAS version 9.2 (SAS Institute, Cary, NC). P value < 0.05 was considered to be significant.
